# Early Evolution of Ionotropic GABA Receptors and Selective Regimes Acting on the Mammalian-Specific Theta and Epsilon Subunits

**DOI:** 10.1371/journal.pone.0000894

**Published:** 2007-09-19

**Authors:** Christopher J. Martyniuk, Stéphane Aris-Brosou, Guy Drouin, Joel Cahn, Vance L. Trudeau

**Affiliations:** 1 Department of Biology and Centre for Advanced Research in Environmental Genomics, University of Ottawa, Ottawa, Ontario, Canada; 2 Department of Mathematics and Statistics, University of Ottawa, Ottawa, Ontario, Canada; University of California at Berkeley, United States of America

## Abstract

**Background:**

The amino acid neurotransmitter GABA is abundant in the central nervous system (CNS) of both invertebrates and vertebrates. Receptors of this neurotransmitter play a key role in important processes such as learning and memory. Yet, little is known about the mode and tempo of evolution of the receptors of this neurotransmitter. Here, we investigate the phylogenetic relationships of GABA receptor subunits across the chordates and detail their mode of evolution among mammals.

**Principal Findings:**

Our analyses support two major monophyletic clades: one clade containing GABA_A_ receptor α, γ, and ε subunits, and another one containing GABA_A_ receptor ρ, β, δ, θ, and π subunits. The presence of GABA receptor subunits from each of the major clades in the *Ciona intestinalis* genome suggests that these ancestral duplication events occurred before the divergence of urochordates. However, while gene divergence proceeded at similar rates on most receptor subunits, we show that the mammalian-specific subunits θ and ε experienced an episode of positive selection and of relaxed constraints, respectively, after the duplication event. Sites putatively under positive selection are placed on a three-dimensional model obtained by homology-modeling.

**Conclusions:**

Our results suggest an early divergence of the GABA receptor subunits, before the split from urochordates. We show that functional changes occurred in the lineages leading to the mammalian-specific subunit θ, and we identify the amino acid sites putatively responsible for the functional divergence. We discuss potential consequences for the evolution of mammals and of their CNS.

## Introduction

Gene duplication followed by gene divergence is one of the major mechanisms responsible for the evolution of new functions [1]. It is thought to underlie the evolution of vertebrates and more particularly their complex and specialized central nervous system (CNS) [2]. Two classes of proteins that underwent such a mechanism are cationic (e.g., acetylcholine, serotonin) and anionic (e.g., glycine, γ-aminobutyric acid) ligand-gated channels, estimated to have diverged before the origin of eukaryotes [3]. However, little more is known about the mode and tempo of evolution of these receptors. Such knowledge may provide us with insights into the structural and functional complexity of these receptors in the central nervous system and in their role in mammalian evolution.

The γ-aminobutyric acid (GABA) is the major inhibitory neurotransmitter found in the vertebrate brain and is involved in CNS development and organization [4], neuroendocrine function [5], and neural processes such as learning and memory [6]. GABA is also present in the nervous system of non-vertebrate taxa, for example, flatworms [7], arthropods [8], [9] and early chordates [10]. GABA synaptic transmission is achieved through membrane bound postsynaptic receptors. Currently, there are three major classes of GABA receptors identified in the mammalian CNS: GABA_A_, GABA_B_ and GABA_C_. These are distinguished according to their composition, pharmacology and localization. Ionotropic GABA_A_ receptors are ligand-gated chloride (Cl^−^) channels consisting of both high abundance subunits (α1-6, β1-4, γ1-3, δ) and low abundance subunits (ε, θ, and π) [11]. Changes in the abundance and composition of these subunits have been shown to induce differences in GABA_A_ receptor sensitivity and response [12], [13]. The current structural model of the GABA_A_ receptor is a pentameric receptor with binding sites for the GABA ligand and for receptor modulation by benzodiazepines, neurosteroids, ethanol, and barbiturates [14]. Metabotropic GABA_B_ receptors are members of the seven transmembrane domain family and are coupled to downstream calcium and potassium channels via G-proteins [15]. Finally, GABA_C_ receptors are also pentameric ionotropic Cl^−^ channels that show similar membrane topology as the GABA_A_ receptors. However, GABA_C_ receptors have unique functional and electrophysiological characteristics, including a slower Cl^−^ conductance and insensitivity to bicuculline and other GABA_A_ receptor modulators [14], [16]. Ionotropic GABA_C_ receptors are composed of ρ subunits that are highly expressed in the vertebrate retina and preferentially localized to bipolar cells [17] but are also found in the spinal cord and pituitary [18]. Both spatial and temporal regulation of GABA receptor subunit expression provide functional diversity to the GABA receptor family.

Fourteen of the human GABA_A_ receptor genes are clustered on four chromosomes (chromosomes 4, 5, 15, and X: [19], [20]). Two of these clusters contain two genes coding for receptor α subunits, one gene coding for a receptor β subunit and one gene coding for a receptor γ subunit whereas the other two clusters contain single genes coding for a receptor α, β and γ subunit–with the ε subunit gene replacing the γ subunit gene on the X chromosome [19]. Evidence based on chromosomal organization, intron/exon structure and transcriptional organization suggests that these four clusters originated from duplications of (and within) an ancestral GABA_A_ receptor gene cluster containing single genes coding for a receptor α, β and γ subunit [19]–[21]. The other two GABA_A_ receptor genes, coding for the receptor δ and the π subunits, also likely arose by duplication of an ancestral GABA_A_ receptor gene(s) but are not part of the four GABA_A_ receptor gene clusters [20]. GABA_C_ receptor genes share only about 35% amino acid sequence identity with GABA_A_ receptor genes [20]. This suggests that these two types of genes may have a common origin and have since acquired distinct functions [20], [22]. However, these studies largely focused on mammalian sequences and the evolutionary history of ionotropic GABA receptor subunits before the origin of mammals is unclear.

Our goal here is twofold. First, we extend our knowledge of the phylogenetic relationships between ionotropic GABA receptor subunits, in particular by including recently available genomes such as those of the sea squirt *Ciona intestinalis* and the pufferfish *Takifugu rubripes*. Second, we test whether positive selection can explain the evolution of the mammalian-specific GABA receptor subunits θ and ε.

## Results

### GABA_A_ receptor phylogeny

**Figure 1:**
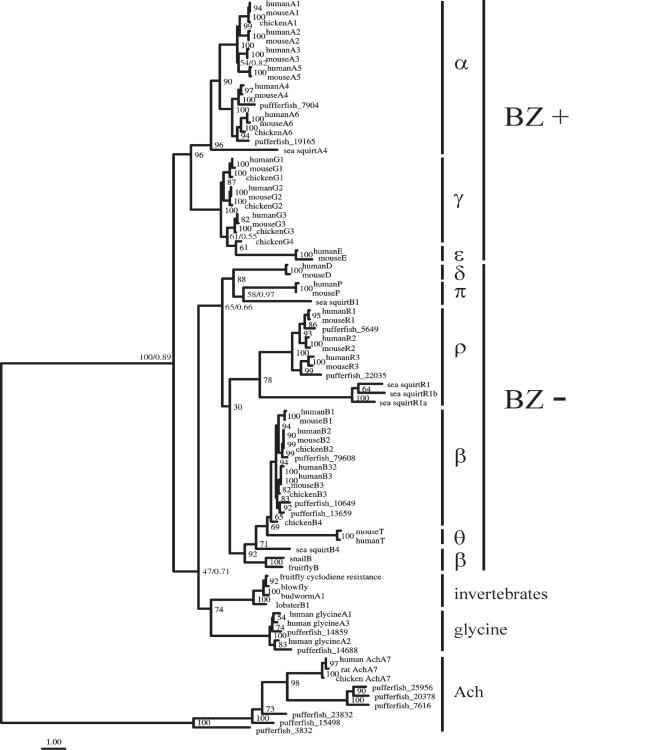
Phylogeny of GABA receptor protein sequences. The two clades, based on the presence (α, γ, and ε) or absence (ρ, β, δ, θ, and π) of a benzodiazepine binding site, are indicated by BZ+ and BZ–, respectively. Bootstrap values (maximum likelihood analysis) are indicated at all nodes while posterior probabilities (Bayesian analysis) are only indicated when smaller than 100%. The scale bar represents 1 amino acid substitutions per site. Ach: acetylcholine receptor.

**Figure 2:**
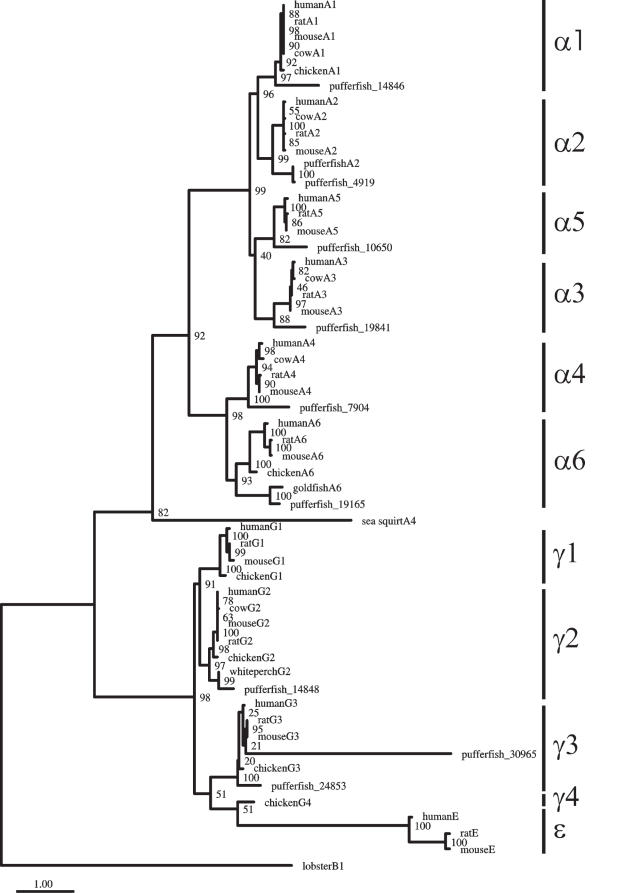
Phylogeny of vertebrate GABA receptor protein sequences with benzodiazepine binding sites. Bootstrap values are indicated at the nodes and the scale bar represents 1 amino acid substitutions per site.

Our estimate of the GABA_A_ receptor phylogeny shows two major monophyletic clades (Fig. 1), which is consistent with previous studies [3], [23]–[25]. This topology is robust to both the search algorithm used and the model of evolution. Indeed, the same topology was obtained by both maximum likelihood and Bayesian approaches, with the latter integrating over different substitution models (where the substitution model used in maximum likelihood [see Methods] had a posterior probability of one). The phylogenetic tree of vertebrate sequences containing the GABA_A_ receptor α, γ, and ε subunits shows that these receptor subunits do not result from non-orthologous gene displacement [26] but appear to be derived from a common ancestor (Fig. 1 and 2). Subunits α are divided into two strongly supported groups: a clade composed of subunits α4 and α6 and the other composed of subunits α1-3 and α5. Subunits γ1 and γ2 form a strongly supported group, whereas the γ3, γ4 and ε subunits all group together. This suggests that the GABA_A_ receptor ε subunits are derived from γ subunits. This is in agreement with previous suggestions that the GABA_A_ receptor subunits γ4 of chicken and ε found in mammals are likely to be orthologous [20], [27].

**Figure 3:**
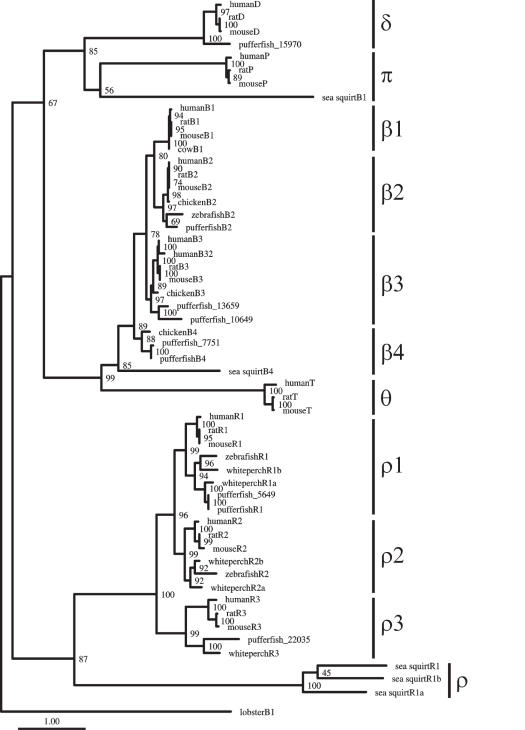
Phylogeny of vertebrate GABA receptor protein sequences without benzodiazepine binding sites. Bootstrap values are indicated at the nodes and the scale bar represents 1 amino acid substitutions per site.

The relationship of the GABA receptor subunits involved in benzodiazepine binding (subunits ρ, β, δ, θ, and π) is uncertain and depends on whether invertebrate GABA/glycine-like receptor sequences are included or not in the analysis. Indeed, when the invertebrate GABA/glycine-like receptor sequences are included (81-sequence data set), subunits δ and π are found to be the sister group to the other GABA receptor sequences in this clade (Fig. 1). In contrast, the tree estimated without the invertebrate GABA/glycine-like receptor sequences (55-sequence data set) shows that subunits ρ are the sister group to other subunit families within this clade (Fig. 3). However and importantly, for both trees, the GABA_C_ receptor ρ subunits, the GABA_A_ receptor θ and β subunits, and the GABA_A_ receptor π and δ subunits, form three strongly supported groups (Fig. 1 and 3).

### Analysis of selective pressures

Our phylogenetic analyses suggested that the θ and ε paralogs have undergone a period of accelerated evolution following the duplication event. Such a period of accelerated evolution could be due to an episode of positive selection affecting these branches. To test this hypothesis, we used a codon substitution model, which measures selective pressures by estimating the nonsynonymous to synonymous rate ratio. This ratio is denoted *ω*, with *ω* = 1, <1 and >1 indicating neutral evolution, purifying and positive selection, respectively [28]. The comparison of a null codon model where selective pressures are constant both along lineages and among sites (H_0_) against a branch-specific or “free-ratio” model (denoted Br in Tables 1 and 2) showed an extensive variation of the *ω* rate ratio among branches in both data sets (Tables 1 and 2).

**Table 1:** Model comparisons and parameter estimates under models of constant (H_0_) or variable *ω* rate ratios across branches (Br), clades (H_1_ to H_3_) or both clades and sites (A and B) for the θ subunit.

Model	*_ℓ_*	H_A_	*P-*value	Parameter estimates	Positively selected sites
H_0_	−8978.84	–	–	*ωˆ* = 0.0521	None
Br	−8903.32	H_0_	<0.0001	–	N/A
H_1_	−8976.36	H_0_	0.0262	*ωˆ*_0_ = 0.0515, *ωˆ*_5_ = 0.4796	N/A
A_0_	−8958.66	–	–	*pˆ*_0_ = 0.46839, *pˆ*_1_ = 0.02186	N/A
				*ωˆ*_5_^(0)^ = 0.04777, *ω*_5_^(1)^ = 1.00000, *ω*_5_^(2)^ = 1.00000	
A	−8952.09	A_0_	0.0003	*pˆ*_0_ = 0.44047, *pˆ*_1_ = 0.02052	(R24N), ({VA}135H), T143C, G224M, Y238R, P253I, M274W, K368P, (M458E), L571E, {DN}605P
				*ωˆ*_5_^(0)^ = 0.04787, *ωˆ*_5_^(1)^ = 1.00000, *ωˆ*_5_^(2)^ = 2.22817	
B_0_	−8861.57	–	–	*pˆ*_0_ = 0.60788, *ωˆ*_0_ = 0.01456_, _ *ωˆ*_1_ = 0.11773	None
B	−8852.74	B_0_	0.0001	*pˆ*_0_ = 0.26659, *pˆ*_1_ = 0.14684	M185L, M274W, M308L, Q363R
				*ωˆ*_5_^(0)^ = 0.01043, *ωˆ*_5_^(1)^ = 0.13544, *ωˆ*_5_^(2)^ = *∞*	
H_2_	−8924.96	H_0_	<0.0001	*ωˆ*_0_ = 0.0149, *ωˆ*_4_ = 0.1597, *ωˆ*_5_ = 0.6079	N/A
H_3_	−8922.42	H_0_	<0.0001	*ωˆ*_0_ = 0.0073, *ωˆ*_1_ = 0.0187, *ωˆ*_2_ = 0.0064	N/A
				*ωˆ*_3_ = 0.0204, *ωˆ*_4_ = 0.1598, *ωˆ*_5_ = 0.6783	

**Figure 4:**
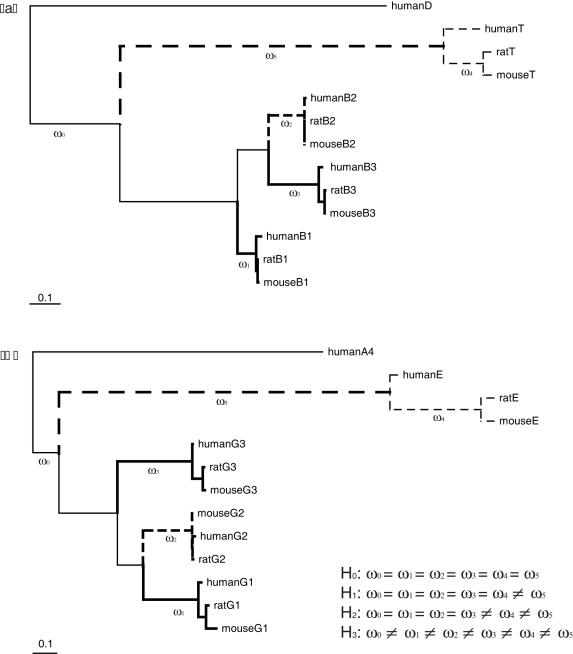
Phylogenetic trees used to formulate hypotheses about the selective forces that shaped the evolution of the GABA_A_ subunits. (a) the GABA_A_ receptor θ and β subunits ; (b) the GABA_A_ receptor ε and γ subunits. Selective pressures are allowed to vary among sets of branches to test for the a priori hypotheses listed at the bottom of the figure: H_0_: the null hypothesis that all branch-specific rates are equal; H_1_: burst of evolution following the main gene duplication event (ε/γ and θ/β); H_2_: also allows for a rate change after the burst of evolution; H_3_: extends H_2_ to allow for burst of evolution after all duplication events depicted on each tree. Hypotheses test are reported in Tables 1 and 2. The scale bar represents 0.1 substitutions per codon site, with branch lengths estimated under the null model (H_0_).

Notes–*ℓ*: log-likelihood value; H_A_: alternative hypothesis to the current model; *ω*_0_: background rate; *ω*_1–5_: branch/clade specific rates as indicated in Figure 4; *p*_0–2_: proportions of sites in each rate category. The hat notation indicates parameters that are free to vary. Positively selected sites were identified with BEB and NEB between brackets for model A and NEB only for model B; a 99% cut-off level of posterior probability was used. Sites putatively under positive selection are numbered according to the human θ reference sequence (accession number BC109210/AAI09211). The underlined sites are those common to both model A and model B. Curly braces indicate equally parsimonious ancestral sites.

**Table 2:** Model comparisons and parameter estimates under models of constant (H_0_) or variable *ω* rate ratios across branches (Br), clades (H_1_ to H_3_) or both clades and sites (A and B) for the ε subunit.

Model	*_ℓ_*	H_A_	*P-*value	Parameter estimates	Positively selected sites
H_0_	−9978.72	–	–	*ωˆ* = 0.0718	None
Br	−9873.60	H_0_	<0.0001	–	N/A
H_1_	−9976.94	H_0_	0.0592	*ωˆ*_0_ = 0.0731, *ωˆ*_5_ = 0.0045	N/A
M1a	−9932.48	–	–	*p*_0_ = 0.88903, *ωˆ*_0_ = 0.06590	N/A
A_0_	−9922.54	–	–	*pˆ*_0_ = 0.72680, *pˆ*_1_ = 0.07894	N/A
				*ωˆ*_5_^(0)^ = 0.06560, *ωˆ*_5_^(1)^ = 1.00000, *ωˆ*_5_^(2)^ = 1.00000	
A	−9921.96	M1a	<0.0001	*pˆ*_0_ = 0.74763, *pˆ*_1_ = 0.08018	{AG}206S, S231K
		A_0_	0.2778	*ωˆ*_5_^(0)^ = 0.06635, *ωˆ*_5_^(1)^ = 1.00000, *ωˆ*_5_^(2)^ = 3.18867	
B_0_	−9788.58	–	–	*pˆ*_0_ = 0.59972, *ωˆ*_0_ = 0.02012, *ωˆ*_1_ = 0.17322	None
B	−9787.81	B_0_	0.4615	*pˆ*_0_ = 0.58359, *pˆ*_1_ = 0.38819	(S231K, S330N, V351C) †
				*ωˆ*_5_^(0)^ = 0.01985, *ωˆ*_5_^(1)^ = 0.17414, *ωˆ*_5_^(2)^ = 1.80401	
H_2_	−9909.20	H_0_	<0.0001	*ωˆ*_0_ = 0.0298, *ωˆ*_4_ = 0.2468, *ωˆ*_5_ = 0.0045	None
H_3_	−9896.02	H_0_	<0.0001	*ωˆ*_0_ = 0.0080, *ωˆ*_1_ = 0.0524, *ωˆ*_2_ = 0.0074	None
				*ωˆ*_3_ = 0.0306, *ωˆ*_4_ = 0.2475, *ωˆ*_5_ = 0.0045	

Notes–*ℓ*: log-likelihood value; H_A_: alternative hypothesis to the current model; *ω*_0_: background rate; *ω*_1–5_: branch/clade specific rates as indicated in Figure 4; *p*_0–2_: proportions of sites in each rate category. The hat notation indicates parameters that are free to vary. Positively selected sites were identified with BEB and NEB between brackets for model A and NEB only for model B; a 95% cut-off level of posterior probability was used. Sites putatively under positive selection are numbered according to the human ε reference sequence (accession numbers HSU66661/AAB49284). †: these sites were putatively identified with a posterior probability >50% (see text). The underlined sites are those common to both model A and model B. Curly braces indicate equally parsimonious ancestral sites.

Our results suggest that the GABA_A_ receptor θ subunit was subjected to positive selection. For reference, we label branches and clades following the conventions set in Figure 4a. When allowing selective pressures to differ specifically in the branch leading to this θ subunit (branch number 5: model H_1_), the model does not significantly explain the data better (at the 1% level) than the constant *ω* rate ratio model (H_0_; Table 1). However, specifically allowing for a different rate in the branch of interest (branch number 5) and allowing *ω* to vary among sites within this branch suggested the existence of sites evolving under positive selection in this branch. Indeed, both the test of positive selection (model A; see Methods and [29]) and its variant (model B; see Methods and [30]) have highly significant *P*-values (Table 1). The estimate of *ω* for the sites allowed to be under positive selection in branch 5 or *ω*_5_^(2)^ under model B (Table 1) is infinity because the synonymous rate along this branch is almost zero. Over all the branches, the average synonymous rate was .356 (excluding root branches), which suggests that saturation may not be an issue for these analyses. However, the sites estimated under models A and B are all different, except for site M274W. While both models consistently detect some evidence for positive selection, the identity of these sites is most likely unreliable, save for the possible exception of site M274W.

**Figure 5:**
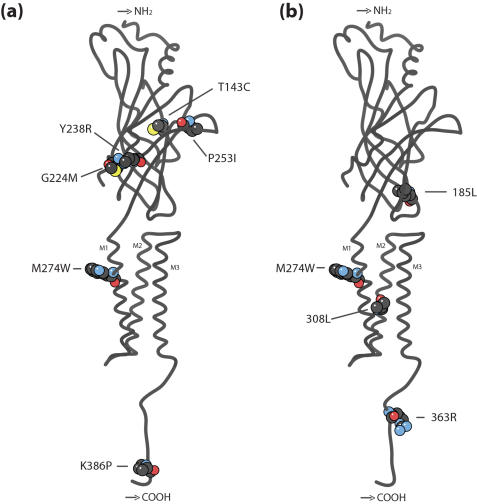
Mapping of the sites putatively under positive selection on the three-dimensional models of the θ subunit of the GABA_A_ receptor. Sites putatively detected to be under positive selection on subunit θ under: (a) model A and (b) model B.

As of July 2007, no three-dimensional (3D) structures for GABA receptor subunits were deposited in the Protein DataBank (PDB: www.pdb.org). Similarity searches based on BLASTp using the protein sequence of the human θ paralog (accession number: AAI09211) returned no structure from PDB. Homology modeling with SWISS-MODEL [31] only produced a model between amino acid positions 291 and 347. This prediction was based on the NMR-obtained template whose PDB accession number is 1VRY, which corresponds to the second and third transmembrane domains of the α-1 subunit of human glycine receptor. The use of recently produced structure of the acetylcholine binding receptor (PDB: 1I9B; [32]) as a template failed to produce any 3D model: this is because the 17% similarity with the human θ paralog is below the 25% threshold of SWISS-MODEL. 3D-JIGSAW [33] produced a larger model that encompasses the previous one and spans from position 62 to 372 (Fig. 5). Because 3D-JIGSAW is more liberal than SWISS-MODEL, its prediction is expected to be somewhat inaccurate outside of the glycine receptor domain. To assess the robustness of our 3D model, we also produced 3D models based on the amino acid sequence of human paralogs β1 (CAA32875), β2 (AAB29370) and β3 (AAA52511). The models produced by 3D-JIGSAW had, after structural alignment, root mean square deviations of 1.21Å, 1.15Å and 1.60Å, respectively, with the model produced with the human θ paralog. These figures are smaller than the 2.5Å resolution usually obtained by X-ray diffraction, so that our model is relatively robust to the protein sequence used within the β/θ group. Although the interpretation of these models should be based on a more rigorous analysis, three domains are apparent on this model (Fig. 5): an N-terminal domain composed of essentially of β-sheets that might correspond to the ligand-binding domain, an intermediate domain composed essentially of α-helices that might correspond to the transmembrane region and a C-terminal domain that might correspond to an intracellular region. This interpretation is consistent with what is known of our model's template (1VRY) and, more generally, of pentameric ligand-gated ion channels such as nAChR, 5-HT_3_, GABA_C_, and glycine receptors [34]. The sites putatively evolving under positive selection along branch 5 are located in all three putative domains of the receptor (Fig. 5a–b). Note that site M274W, identified by both model A and model B is located within the putative transmembrane region on the first α-helices (M1: Fig.5; [34]). Further analyses allowing *ω* to differ among branches leading to the β paralogs (models H_2_ and H_3_) are consistent with an episode of positive selection leading to the θ paralogs, followed by a regime of negative selection. To summarize, evidence for positive selection acting in this branch is strong, seems to have affected all three putative domains of the receptor subunit, but the identification of the actual sites should be taken with caution due to the relatively small number of sequences analyzed here.

Results from the ε data set only show evidence for relaxed selective pressures along the branch leading to the ε subunit. Here also, branches and clades are numbered following conventions in Figure 4b. As shown above, the reconstruction of this region of the GABA tree is unstable (Fig. 1 and 2). However, all results shown below were identical to the fifth decimal place under both tree reconstructions. This probably reflects (i) the low support for the ε clade (Fig. 1 and 2) and (ii) the relative robustness of site codon models to the exact topology of the tree as long as this tree is reasonable [35], [36]. As with subunit θ, the fit of model H_1_ is not significantly better than that of the one ratio model (H_0_), but neither test with model A or with model B are significant (Table 2). Importantly, the comparison of model A with a model called M1a [37] that assumes only two classes of sites, with 0<*ω*<0 and *ω* = 1, is highly significant (Table 2). This latest comparison, called “test 1” in [29], cannot discriminate efficiently between positive selection and relaxed selective pressures [29]. Taken together, a significant “test 1” and a non-significant “test 2” suggest that the ε subunit only underwent relaxed constraints. Because of the conservative nature of “test 2” (see Methods and [29]), and because the average synonymous rate was 4.105 (excluding root branches) in the ε/γ data set, more sequences will be required to definitely rule out the action of positive selection during the evolution of the ε subunit.

## Discussion

The present analyses and previous work [3], [23] all suggest that an ancestral GABA-like receptor subunit gave rise to two monophyletic clades, categorized as subunits involved (α, γ, and ε) or not involved (ρ, β, δ, θ, and π) in benzodiazepine binding. As previously found [24], [25], [38], the presence of putative GABA α-, β- and ρ-like receptor subunits in the genome of *C. intestinalis* support that this ancestral duplication most likely occurred before the divergence of urochordates. These results suggest that benzodiazepine sensitivity evolved early, in marked contrast to previous proposals [39], [40]. In line with our finding, recent electrophysiological data suggest that invertebrates (e.g., *Hydra vulgaris*) are responsive to benzodiazepine modulation and this response is similar to the response to GABA [41]. On the other hand, the presence of α and β-like subunits in *C. intestinalis* does not necessarily indicate benzodiazepine sensitivity.

It was previously suggested that the GABA_A_ receptor δ subunit is the most primitive subunit within the GABA_A_ clade not involved in benzodiazepine binding [3]. Our phylogenetic analyses were unable to confidently determine the probable progenitor for receptor subunits in this clade (Fig. 1 and 3). Within the GABA_A_ receptor β subunit clade, it is interesting to note that our genome searches did not identify any β1 sequences in pufferfish or chicken genomes. This suggests that the pufferfish and chicken lineages may have independently lost their β1 paralog.

Our phylogenetic analyses also indicate that the mammalian-specific GABA_A_ subunits ε are derived from the γ4 subunit (Fig. 1 and 2), which is consistent with their chromosomal organization: Human GABA_A_ receptor ε genes are positioned on a location of the X chromosome that corresponds to the position of GABA_A_ receptor γ4 genes on human chromosomes 4, 5 and 15 [19], [20], [27]. The higher rate of evolution of the GABA_A_ receptor ε subunits may explain why these subunits have so far only been found in mammals while the receptor γ4 subunits are present in both birds and reptiles [20]. Similarly, chromosomal location [20], intron-exon organization [21] and our phylogenetic analyses are consistent with the suggestion that the mammalian-specific GABA_A_ receptor θ subunits are derived from GABA_A_ receptor β4 subunits (Fig. 1 and 3).

Although we could only identify a small number of GABA_A_ receptor sequences from the *C. intestinalis* genome, the pufferfish genome contains orthologs to most of the GABA_A_ receptor sequences found in mammalian genomes. Furthermore, all the vertebrate genomes queried, including that of pufferfish, contain a single gene coding for most of the 20 GABA_A_ receptor subunits (α1-6, γ1-3, ε, δ, π, ρ1-3, β1-4 and θ). However in pufferfish, five of these gene families contain two paralogous gene copies instead of a single copy. Three of these pairs (pufferfish α2, β4 and ρ1) are composed of very similar genes that have undergone recent duplication events. It would be interesting to test experimentally whether the subunits encoded by these gene copies have similar functions. Examples of recent gene duplication events show that it is not necessarily the case: receptors for proglucagon-derived peptides for instance exhibit differences in ligand-binding capabilities between recent paralogs [42]. The presence of two paralogous gene copies in some of the GABA_A_ receptor subunit families found in the pufferfish genome is consistent with a complete genome duplication event that occurred in the pufferfish lineage [43], [44]. Further studies should elucidate whether duplicated GABA receptor subunits have a functional significance for GABAergic transmission in fish.

Our codon analyses showed that both GABA_A_ receptor subunits θ and ε experienced positive selection and relaxed constraints, respectively (Table 1–2). We further identified the putative amino acid sites that may be responsible for the functional divergence of subunit θ (Table 1 and Fig. 5). One position in subunit θ (274) was consistently identified as being under positive selection by different models. This position is located in putative transmenbrane segment M1, which, like M3, is though to interact with their neighboring subunits in the pentameric receptor [34]. The functional significance both of this location and of the adaptive substitution by a larger and aromatic residue at this position in subunit θ are unclear. Yet, positive selection acting on the sites in the N-terminal domain might have affected the ligand-binding affinity of GABA agonists and or antagonists, and maybe also allosteric transitions between different conformational states, that are the functions associated to this domain in nicotinic receptors [34]. On the other hand, positive selection acting on the C-terminal domain might have affected the sensitivity of the receptor, a property that is determined by the five loops constituting the pentameric receptor, at least in nicotinic receptors [34].

Relaxed constraints in the ε subunit would be consistent with the observation that rodent ε subunit have acquired an unusually large insertion of 483 amino acids in their second exon [27]. These subunits are expressed in the CNS, and are less abundant than other subunits [11]. In rats, the expression of ε subunits is associated with peptidergic neurons, such as those producing orexin, oxytocin, and gonadotropin-releasing hormone (GnRH). This suggests that GABA_A_ receptors with ε subunits might have a role in neuroendocrine function, such as that involved in the control of feeding and reproduction [45]. A recent study showed that ε subunits could increase GABA sensitivity up to 100-fold in *Xenopus* oocytes [46]. It was also shown that receptors containing the ε subunit could be insensitive to the GABA receptor modulators pregnanolone and pentobarbital [46]. Interestingly, these GABA receptor subtypes are predominant in the locus coeruleus, a nucleus in the brain stem that contains a large population of noradrenergic positive neurons. When this region is lesioned in rats, there is a disruption in the preovulatory surge of luteinizing hormone (LH), follicle-stimulating hormone (FSH), and prolactin [47] and a significant reduction in circulating plasma LH [48]. Further studies should assess the role of the GABA_A_ receptor θ and ε subunits in this neuroendocrine pathway. Should these subunits be involved in this pathway, our results would suggest that the rapid divergence of the GABA_A_ receptor θ and ε subunits played a role in the evolution of the neuroendocrine system in mammals. Recent studies utilizing point-mutations have shown that single amino acid changes in a GABA receptor subunit will have dramatic effects on the kinetics of the receptor [49], [50]. Future studies are needed to determine whether or not these amino acid changes confer significant alterations in GABA receptor kinetics and function.

### Conclusions

To conclude, our results show that (1) the two major clades of ionotropic GABA receptors arose before the split from urochordates, (2) the GABA_A_ receptor family evolved by both gains and losses of subtypes (e.g., teleost β4, chicken γ4, mammalian ε and θ) and (3) the function of the GABA receptor subunits might have changed adaptively in the mammalian-specific GABA_A_ subunit θ, while relaxed constraints acted on subunit ε. These changes of selective regime might have played a role in the evolution of neuroendocrine functions controlling feeding and reproduction in mammals. We caution however that further research should be performed to experimentally test these functional divergence hypotheses.

## Materials and Methods

### Genome database searches

Homologous gene queries were performed on the National Center for Biotechnology Information (NCBI; http://www.ncbi.nlm.nih.gov) server. BLASTn and BLASTp searches [51] were used to find homologous genes of full-length GABA receptor subunits α1-6, β1-3, γ1-3, δ, ε, θ, and π present in the completed rat genome project. The *Ciona intestinalis* sequences were obtained from the Joint Genome Institute (http://genome.jgipsf.org/ciona4/ciona4.home.html). Sequence alignments are available as supplementary Text S1 and S2.

### GABA receptor phylogeny

We obtained and aligned a data set of 81 homologous protein sequences of the GABA receptor subunits queried above. This data set contains almost exclusively complete protein sequences with 997 aligned amino acid positions; this alignment is much longer than the actual length of GABA receptors (ca. 470 amino acids) because of the presence of large indels in some specific subunits. All aligned positions were conserved to help tease apart highly conserved proteins. To test for robustness of these analyses, we increased the species sampling of receptors involved in benzodiazepine binding (BZ+: α, γ, ε) and those that are not (BZ–: ρ, β, δ, θ, and π) [3], [23]. Note that this BZ+/BZ– classification of subunits is somewhat artificial as two subunits are actually required to form a benzodiazepine sites [11]. We sampled 55 chordates including sea squirt and vertebrates for the BZ+ GABA_A_ receptor protein sequences (1,007 aligned amino acid positions) and 55 chordates including sea squirt and vertebrate BZ– protein sequences (795 aligned amino acid positions). Again, discrepancies between actual protein lengths and length of the alignment reflect the presence of indels.

Phylogenetic trees were inferred using both the maximum likelihood approach as implemented in PHYML 2.4.4 [52] and the Bayesian approach implemented in MrBayes 3.1.1 [53]. In both approaches, the 81-sequence data set was rooted with acetylcholine receptor protein sequences [3], [23]. Based on the results from the analysis of this data set (Fig. 1), the BZ+ and BZ– data sets (Fig. 2 and 3, respectively) were rooted with the lobster β1 (accession number AY098945) because this sequence provides a closer outgroup to both data sets (and that closer outgroups minimize possible errors in phylogenetic trees). ProtTest [54] was used to determine, based on the Akaike Information Criterion (min*AIC*), that the JTT+I+Γ model of amino acid substitution [55], [56] was the most appropriate model of evolution for this data set (*pˆ_I_* = 0.01; *αˆ* = 0.819); the second to most appropriate model was JTT+Γ (Δ*AIC* = 48.81), while the second to most appropriate rate matrix was WAG (+I+Γ: Δ*AIC* = 444.74; +Γ: Δ*AIC* = 523.11). Among-site rate variation modeled by a discrete Γ distribution with eight rate categories. This maximum likelihood model was fitted independently to each data set.

Because model choice as performed by ProtTest can lead to underestimating uncertainty, the maximum likelihood analyses were complemented by a Bayesian analysis that integrates over different models of evolution: rather than selecting an a priori empirical model of substitution, a reversible-jump Markov chain Monte Carlo (RJ-MCMC) was constructed to integrate over model uncertainty [57]. Models with equal prior probability were: Poisson, JTT, Dayhoff, MtREV, MtMAMM, WAG, rtREV, CpREV, VT and BLOSUM62 as described and implemented in MrBayes [53]. Among-site rate variation was modeled using a discrete Γ distribution with five rate categories [56] plus a class of invariable sites. Under this mixed model of protein evolution, four independent RJ-MCMC samplers were run for ten million steps using different starting values. To decrease autocorrelation of the samples taken from the target distribution, steps along the chain were sampled every 1,000 accepted steps, a method known as thinning (e.g., [58]). To improve mixing, each sampler consisted of four chains, three of which were heated to different temperatures (e.g., [56]). By raising the likelihood function to a power <1, deep valleys of the likelihood surface become shallow, which facilitates their crossing by the sampler and hence improves the ability of the chain to explore the entire parameter space efficiently, in proportion of the target density. Sampling was realized from the non-heated chain. Burn-in length and convergence of the samplers were checked by plotting time series plots and checking that average standard deviations of split frequencies were lower than 0.01 [53]. The chains appeared to have converged by 10,000 steps; to be conservative, 100,000 steps were discarded as a burn-in.

### Analysis of selective pressures

Codon data were obtained and split into two smaller data sets, one for each of the paralogous clade of interest: θ and ε. Each data set contained 13 sequences human (*Homo sapiens*), rat (*Rattus norvegicus*) and mouse (*Mus musculus*) copies of θ and ε paralogs, as well as the corresponding members of the group in which this clade was located: β for θ; γ for ε. Each tree was rooted by the closest human paralog (δ and α, respectively). Sites with ambiguous data were removed. A statistical approach was then used to detect functional divergence at individual codon sites within the pre-specified branches based on a procedure similar to that by Bielawski and Yang [59]: for each paralog the null hypothesis H_0_ was that of no variable selective pressure among branches and among sites (Fig. 4). This hypothesis was contrasted by means of likelihood ratio tests against three potential alternative modes of evolution. In H_1_, only the branch leading to the θ or to the ε clade was allowed to evolve at a different *ω* rate ratio. This branch (branch number five) is said to have a foreground rate, while all the other branches have the same background rate. In a second model, H_2_, the θ or the ε clade was allowed to evolve at a rate that differs from both the foreground and the background rates. Finally in H_3_, each paralog was allowed to evolve at its own rate after duplication.

In some cases, only a few sites are affected by an episode of positive selection within a given branch. A more powerful approach to detecting sites undergoing positive selection in such cases is to allow *ω* to vary among sites within the branch of interest using the “test of positive selection” or “test 2” described in [29] and implemented in PAML version 3.15 [60]. These “branch-site” models allow the *ω* ratio to vary both among branches and among the sites in the foreground branch. Model A has four classes of sites: class 0 includes conserved codons with 0<*ω*_5_^(0)^<1 is estimated from the data; class 1 includes codons that evolve neutrally (*ω*_5_^(1)^ = 1); classes 2a and 2b include codons that are conserved or neutral on the background branches, but are under positive selection on the foreground branch, with *ω*_5_^(2)^>1 estimated from the data. The “test of positive selection” compares this model against a simpler (null) model, that does not allow for positive selection (*ω*_5_^(2)^ = 1), so that this model estimates one fewer parameters than its alternative. To be conservative, we used *χ*_1_^2^ as an approximation to the distribution of the test statistic under the null [29]. Robustness of these branch-site codon models was assessed by using a second test based on “model B” as described in [29], which is identical to model A described above except that *ω*_5_^(1)^ is free to vary. Model B is compared to model M3 [35] which is a site model with two discrete rate categories. Sites putatively under adaptive evolution in the “test of positive selection” were identified by the Bayes empirical Bayes (BEB) procedure [37], that improves on the naïve empirical Bayes approach (NEB: [61]) by accommodating uncertainties of the maximum likelihood estimates; with “model B”, only BEB is implemented to assign individual sites to rate categories. All codon models were run at least twice to check convergence. Ancestral amino acid residues were determined by parsimony (for Tables 1 and 2).

Three-dimensional (3D) structure predictions were carried out with 3D-JIGSAW [33] and with SWISS-MODEL [31]. Both tools predict structures by homology modeling, a technique that can be decomposed into five steps: (i) a query or parts thereof are aligned to one or more template protein sequences, as determined by BLASTp searches; templates must have a resolved 3D structure; (ii) these 3D segments are put together to form a preliminary model; (iii) side chains are adjusted to account for substitutions between the query and the templates; (iv) the model is examined for potential collisions between atoms; finally (v) the model is refined by limited energy minimization. The major inaccuracies of homology modeling usually stem from two sources: low sequence similarity and improper template selection [62]. These two sources of inaccuracies were assessed as explained in the text. Structural alignments of the backbones of the models and computation of root mean square deviations or RMSDs were performed with DeepView, which is available through the ExPASy Web site.

## Supporting Information

Text S1 Amino acid alignment of the 81 full-length GABA receptor subunits.
(0.08 MB TXT)

Text S2 Codon alignment of the GABA receptor subunits β, θ, γ and ɛ for human.
(0.09 MB TXT)
